# Identification of Distinct Heterogenic Subtypes and Molecular Signatures Associated with African Ancestry in Triple Negative Breast Cancer Using Quantified Genetic Ancestry Models in Admixed Race Populations

**DOI:** 10.3390/cancers12051220

**Published:** 2020-05-13

**Authors:** Melissa Davis, Rachel Martini, Lisa Newman, Olivier Elemento, Jason White, Akanksha Verma, Indrani Datta, Indra Adrianto, Yalei Chen, Kevin Gardner, Hyung-Gyoon Kim, Windy D. Colomb, Isam-Eldin Eltoum, Andra R. Frost, William E. Grizzle, Andrea Sboner, Upender Manne, Clayton Yates

**Affiliations:** 1 Department of Surgery, Weill Cornell Medicine, New York, NY 10065, USA; mbd4001@med.cornell.edu (M.D.); rnm4001@med.cornell.edu (R.M.); lan4002@med.cornell.edu (L.N.); 2 Department of Physiology and Biophysics, Weill Cornell Medicine, New York, NY 10065, USA; ole2001@med.cornell.edu; 3 Caryl and Israel Englander Institute for Precision Medicine, Weill Cornell Medicine, New York, NY 10065, USA; ans2077@med.cornell.edu; 4 Institute for Computational Biomedicine, Weill Cornell Medicine, New York, NY 10065, USA; 5 Department of Biology and Center for Cancer Research, Tuskegee University, Tuskegee, AL 36088, USA; jwhite7264@tuskegee.edu (J.W.); wdeancol@gmail.com (W.D.C.); 6 Department of Computational Biology, Weill Cornell Medicine, New York, NY 10065, USA; akv3001@med.cornell.edu; 7 Department of Public Health Sciences, Henry Ford Health System, Detroit, MI 48202, USA; idatta1@hfhs.org (I.D.); iadrian1@hfhs.org (I.A.); ychen4@hfhs.org (Y.C.); 8 Department of Pathology and Cell Biology, Columbia University, New York, NY 10027, USA; klg2160@cumc.columbia.edu; 9 Department of Pathology, University of Alabama at Birmingham, Birmingham, AL 35233, USA; hgkim@uabmc.edu (H.-G.K.); ieltoum@uabmc.edu (I.-E.E.); afrost@uabmc.edu (A.R.F.); wgrizzle@uabmc.edu (W.E.G.); 10 Department of Hematology and Oncology, Our Lady of Lourdes JD Moncus Cancer Center, Lafayette, LA 70508, USA; 11 O’Neal Comprehensive Cancer Center, University of Alabama at Birmingham, Birmingham, AL 35233, USA; 12 Department of Surgery, University of Alabama at Birmingham, Birmingham, AL 35233, USA; 13 Department of Pathology and Laboratory Medicine, Weill Cornell Medicine, New York, NY 10062, USA

**Keywords:** triple negative breast cancer, African ancestry, RNAseq analysis, oncologic pathways, disparities

## Abstract

Triple negative breast cancers (TNBCs) are molecularly heterogeneous, and the link between their aggressiveness with African ancestry is not established. We investigated primary TNBCs for gene expression among self-reported race (SRR) groups of African American (AA, *n* = 42) and European American (EA, *n* = 33) women. RNA sequencing data were analyzed to measure changes in genome-wide expression, and we utilized logistic regressions to identify ancestry-associated gene expression signatures. Using SNVs identified from our RNA sequencing data, global ancestry was estimated. We identified 156 African ancestry-associated genes and found that, compared to SRR, quantitative genetic analysis was a more robust method to identify racial/ethnic-specific genes that were differentially expressed. A subset of African ancestry-specific genes that were upregulated in TNBCs of our AA patients were validated in TCGA data. In AA patients, there was a higher incidence of basal-like two tumors and altered TP53, NFB1, and AKT pathways. The distinct distribution of TNBC subtypes and altered oncologic pathways show that the ethnic variations in TNBCs are driven by shared genetic ancestry. Thus, to appreciate the molecular diversity of TNBCs, tumors from patients of various ancestral origins should be evaluated.

## 1. Introduction

According to national surveillance data for the United States (US), non-white minority populations suffer higher mortality rates for most cancers [1]. This has largely been considered a consequence of poor health care equity and/or access [2,3] related to the prevalence of lower socioeconomic status (SES) for minority populations. However, European Americans (EAs) have historically been diagnosed with a higher incidence of breast cancer, compared to African Americans (AAs). Prior to the mid-1980s, breast cancer mortality rates for these self-reported race (SRR) groups was essentially the same, but then diverged in subsequent years. These persistent survival disparities are currently about 40% [1,4] and occur independent of SES, which suggests there are additional factors, including biology, leading to race-group differences in mortality. 

The onset of race-group mortality rate disparities coincides with the advent of hormone-targeted therapies [5] that are now standard-of-care for hormone receptor-positive tumors. Compared to women of European descent, AA women [4,6,7,8,9,10,11,12,13] and women of African descent world-wide [9,14,15,16] have a higher incidence of triple-negative breast cancer (TNBC) [17,18,19,20,21], which is characterized by the absence of estrogen receptor (ER), progesterone receptor (PR) and human epidermal growth factor receptor 2 (HER2). Therefore, in the context of standardizing ER/PR- and HER2-targeted therapies, the divergence of AA vs. EA mortality likely unmasked population-level differences in tumor biology, which we have previously shown to correlate with genetic ancestry [22]. Several epidemiological studies suggest that genetic ancestry is a factor in the etiology of specific tumor phenotypes [13,23,24,25], with disease outcomes based upon molecular phenotype (e.g., HR status) directly affecting treatment decisions, regardless of SES barriers to high-quality clinical care.

TNBC, one of the most aggressive forms of breast cancer, has limited treatment options that are ineffective when the cancer is diagnosed at later stages [25,26,27,28,29]. Since AA women tend to be diagnosed at later stages [30,31], at an early age [32,33,34], and suffer higher rates of TNBC, these factors likely contribute to AAs having the highest breast cancer mortality rate among all race groups. Even within TNBC cases, AA women have a higher mortality compared to EA women [4], and these race-/ethnicity-associated differences in TNBC survival suggest that there is a difference in disease progression, which may be driven by differences in gene expression that are detectable by genomic investigations. Multiple lines of evidence support this theory, including differences in the prevalence of “*Vanderbilt TNBC subtypes*” [35,36] among SRR groups, in which gene expression signatures define these subtypes. Although this TNBC subtype classification was intended to assist with clinical management and identification of targetable genes in TNBCs [36], these subtypes represent a myriad of heterogeneity [37,38] that has yet to be fully defined for understudied/minority populations, who suffer most from TNBC.

To have better representation of phenotypic variation in TNBC tumors, we report here our investigation of differences in TNBC primary tumor gene expression using bulk RNAseq, comparing TNBCs of AAs to those of EAs. As opposed to use of traditional methods identifying differentially expressed genes (DEGs) between SRR groups, we quantified genetic ancestry (QGA) for individual patients across five human ancestry super groups, and identified the African ancestry-associated gene expression signatures of TNBCs. We then determined whether these racial/ethnic differences in gene expression reveal insights into biological pathways, and characterized the TNBC subtypes using a newly revised method for categorizing subtypes, building upon previously validated tools. We also characterized tumor-associated immune responses for each tumor. Furthermore, we determined whether phenotypic subtypes were associated with genetic ancestry, as well as whether there were biases of prevalence of TNBC phenotypes between patient SRR and ancestry groups. 

## 2. Results

### 2.1. African Ancestry-Associated Gene Signatures in Treatment-Naïve and Post-Treatment TNBCs

**Figure 1:**
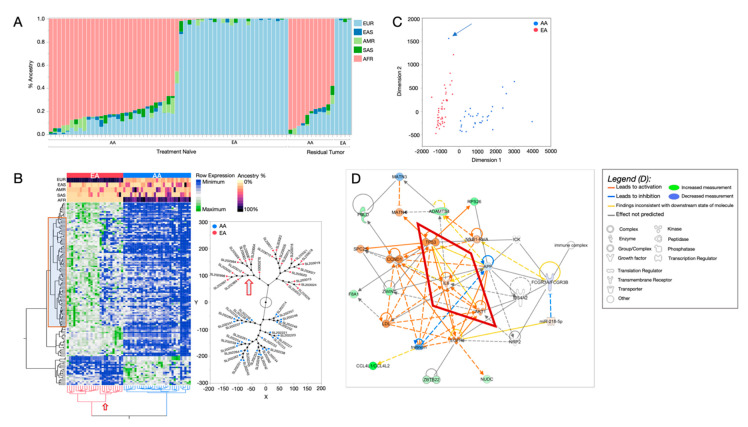
Differentially expressed genes (DEGs) associated with quantified genetic ancestry (QGA) in treatment-naïve TNBC RNA-seq. (**A**) QGA estimates for each cancer case, derived from RNAseq variants. Geographic ancestry super-group categories are indicated as European (EUR, light blue), East Asian (EAS, dark blue), American Native (AMR, light green), South Asian (SAS, dark green), and African (AFR, pink). Samples are grouped by treatment status (treatment naïve or residual tumor) and self-reported race (SRR). (**B**) Clustergram heatmap of the 156 (*p* < 0.05) genes that show differential expression levels by QGA, where rows represent genes and columns represent individuals. SRR is shown in the top row of the color map (red indicating EA, and blue indicates AA); the remaining color map rows indicate ancestry estimations for each individual. The red box indicates genes that are associated with non-European admixture (EAS, SAS, and AMR). Constellation plot, right, representing the hierarchical structure of the individuals shown at the bottom of the heatmap. Red dots indicate SRR EAs; blue dots are SRR AAs. The red arrow points to the substrata of EA individuals with increased admixture; this corresponds to the non-European admixture genes in the red box of the heatmap. (**C**) Multidimensional analysis using 156 ancestry-associated genes indicates that the expression patterns separate individuals into SRR groups. Red indicates EA, and blue indicates AA. The blue arrow indicates an individual that self-reported as EA but has mostly AFR ancestry, clustering with the AA group. (**D**) De novo network analysis using QGA DEGs using Ingenuity Pathway Analysis (IPA) software. Molecules in green are upregulated in individuals with increased AFR ancestry; those in blue are downregulated in individuals with AFR ancestry. Molecules in orange are drawn into the network and predicted to be activated based on the state of DEGs in the network, using published interactions from the curated Ingenuity Knowledge Base. Orange lines between molecules indicate relationships leading to activation; blue lines indicate relationships leading to inhibition. Yellow lines indicate that the relationship between two molecules is not in the expected direction. For example, in this network TP53 is known to inhibit AKT1. TP53 is activated, and so it is expected that AKT1 would be downregulated. However, it is not. Because of this, the line showing the interaction between these two molecules is shown as yellow.

In an effort to uncover differentially expressed genes that are driven by shared ancestry, and therefore presumably under distinct genetic regulation, we first quantified the individual genetic ancestry of our cohort across five human super-groups. The initial step for this ancestry estimation included identification of SNVs from the bulk RNAseq data. These variants were compared to the 1000 Genomes super-group reference sets to estimate proportional ancestry and correlated to the 1000 Genomes populations. Specifically, each individual was measured for European [39], East Asian (EAS), South Asian (SAS), American Native (AMR), and African (AFR) ancestry (Figure 1A). As expected, most genetic ancestry for EAs was European (86–99%), and most ancestry for AAs was African (45–98%). However, a portion of the EA patients had appreciable Asian and/or American Native admixture (2–15%). Similarly, most AA patients had substantial European (0–44%) or American Native (0–14%) ancestry. This analysis also revealed that, based on the molecular signature of their tumors, two patients who self-reported as EAs had more than 60% of African ancestry and clustered with AA patients.

Using the QGA for each individual, we conducted gene-by-gene linear regressions, screening for significant associations with each of the QGA super-groups. Among all ancestry group tests, AFR and European ancestry estimations yielded the largest and most significant set of genes with ancestry-associated expression changes, compared to EAS, SAS, and AMR. For the treatment-naïve tumors (31 AAs and 29 EAs), 156 genes were significantly associated with African ancestry (adjusted *p*-value < 0.05, Table S4). Similarly, we conducted a QGA-association analysis on 15 post-treatment tumors (residual tumors), but low patient numbers (11 AAs and four EAs) impeded our ability to reach statistical significance (adjusted *p*-value threshold < 0.05). Alternatively, we accomplished a traditional SRR group comparison and found 13 SRR-associated genes in residual tumors that were not identified in our SRR treatment-naïve analysis (adjusted *p*-value < 0.05) (Figure S2). 

Most ancestry-associated genes showed a negative correlation in expression (downregulation) for those of African ancestry patients compared to those of European ancestry (Figure 1B). In a two-way hierarchical clustering of both gene expression and patient samples, SRR groups arbitrarily clustered together (Figure 1B). Further, when we investigated the phylogenic structure of the patient cluster nodes, we found that EA patients separated into two distinct groups (Figure 1B, red arrow). The separate EA groups had differences in gene expression patterns as well as genetic ancestry composition, with one group primarily containing only European ancestry, and the second group containing a substantial amount of genetic admixture from EAS, SAS, and AMR. The gene expression patterns of the admixed EAs were similar to the AA group (Figure 1B), which also had substantial EAS, SAS, and AMR admixture. Therefore, this separation of EA patients provided further evidence of the impact of genetic ancestry on gene expression. Using this gene set, a principal component analysis (Figure 1C) with the African ancestry-associated genes completely segregated the SRR groups, suggesting that this gene signature from TNBCs predicts race/ancestry among TNBC patients. Because these genes were selected for their association with African ancestry, and ancestry estimates were highly correlated with self-identity, we are confident that this set of genes is representative of genes that are distinctly regulated among race/ethnicity groups, due to individual-level African ancestry.

Since several EA patients had no appreciable African ancestry, we also determined the association of European ancestry with gene expression, using the same linear regression model used for the African-associated gene selection. As a way of validating the method’s capacity to identify ancestry-associated genes, we noted whether the genes associated with European ancestry were unique, compared to genes associated with African ancestry. Of the 156 African-associated genes, 153 overlapped with genes associated with European ancestry; seven additional genes were European-specific and the remaining three were African-specific (Figure S1C). For the genes shared between African and European ancestry, trends of gene expression positively correlated with African ancestry, but negatively correlated with European ancestry. This contrast suggests that ancestral informative alleles that are population-private (i.e., existing in one ancestry group and not the other) are the genetic drivers regulating gene expression levels.

We also determined differences in expression comparing SRR groups of EAs with AAs in order to compare the results of our genetic ancestry method to the traditional race-group comparison method (Figure S3). The SRR DEG comparison yielded more than 1000 genes (Figure S3A) with significant differential expression (adjusted *p*-value < 0.05; upregulated genes = 266, downregulated genes = 758, Table S5.). However, hierarchical clustering revealed that the range of gene expression differences for the SRR DEGs was smaller compared to the QGA-associated genes, translating to higher absolute fold changes in QGA-associated genes (avg∆ = 2.4) compared to SRR DEGs (avg∆ = 1.14) (Figure S3C). Compared to the ancestry-associated genes, 81 genes were shared between SRR DEG analysis and QGA DEG analysis. This suggests that, when comparing SRR groups, less than 8% of the DEGs will be due to genetic ancestry; the remaining 92% could be due to socio-clinical factors (e.g., comorbidity, environmental exposures).

### 2.2. Distinct Biological Networks of African Ancestry and Differentially Expressed Genes

We investigated whether genes that show expression changes associated with African ancestry are involved in biological pathways that could suggest distinct ancestry-specific functionality. We calculated the fold-change differences for the 156 African-associated genes between the SRR race groups, EA and AA, for pathway enrichment analysis. For network predictions, we used Ingenuity Pathway Analysis (IPA) software [40] (Figure 1D). We conducted a causal network analysis, which assessed known connections across all African-associated genes to predict how these interactions may have been altered, based on gene expression changes between the SRR groups. The most prominent network was derived from 25 genes that were associated with African ancestry, with an additional 10 genes that were automatically included through knowledgebase predictions, based on previously published interactions. In the de novo network, canonical cancer-related pathways, including NfKB, TP53, and EGFR, were involved and predicted to be activated among AA individuals (Figure 1D). Several interactions within the network were designated “inconsistent” in the context of the established expected gene regulation effects. An example of these unexpected interactions is central to understanding how ancestry influences these networks (Figure 1D, red box). For example, TP53 is predicted to be activated in AAs based on gene expression being higher; however, the expected/established outcome of TP53 activation would be down-regulation of the AKT1 kinase and IL6 chemokine genes. However, in our ancestry-related pathway analysis, both genes appeared to be activated when TP53 was activated. Hence, the African-ancestry associated genes had altered expression, leading to unexpected relationships inconsistent with previous findings. We also determined the general pathway enrichment among the African-associated genes; the most significantly enriched pathways are shown in Figure S1C. 

An additional pathway analysis, utilizing DEGs from the SRR comparison (Figure S2), also revealed involvement of canonical cancer-related pathways. As seen in Figure S2E, the top gene ontology diseases and functions in the de novo system were behavior, cellular assembly and organization, and connective tissue disorders. A canonical cancer pathway involving the NFkB complex is central to this network (Figure S2E), which is predicted to activate (denoted by orange relationship lines) various genes upregulated in the dataset, including the transcription regulator CITED4, peptidase ADAMTS9, and kinase PIM3. 

### 2.3. Prevalence of TNBC Subtypes across Race and Ancestry Groups

**Figure 2:**
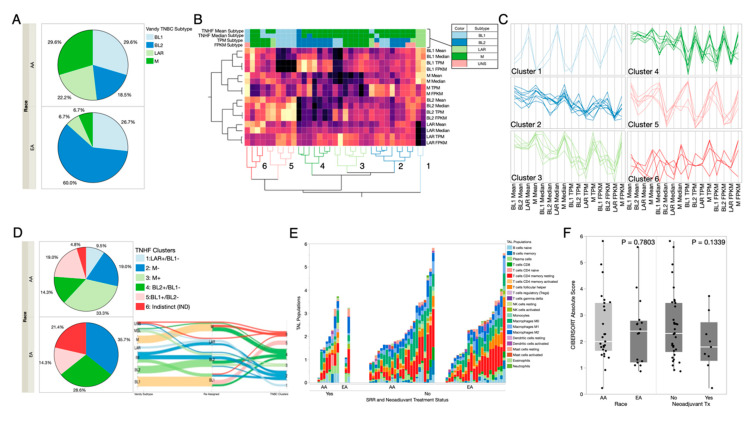
TNBC subtyping and distribution among SRR and treatment groups. (**A**) Distribution of re-assigned TNBC subtype calls across SRR race groups using the Vanderbilt calling method. (**B**) Clustergram heatmap of TNBCType call correlations for BL1, BL2, LAR, and M subtypes from use of the Vanderbilt tool with TPM (transcripts per million) and FPKM (fragments per kilobase of exon model per million reads mapped) values as input and our TNBC subtyping method (TNHF mean and median). Rows represent the different subtype correlations for the tools, and columns represent individuals. Using these correlations, our samples separate into six clusters, numbered at the bottom. Color map columns of the call reassignments removing IM and MSL are shown at the top of the heatmap (key to the upper right). Sample names are colored based on their cluster assignment. Reassignment of TNBC subtypes based on a dual-tool reduction method. Cluster Nodes: 1 = LAR+/BL1−, 2 = M−, 3 = M+, 4 = BL2+/BL1−, 5 = BL1+/BL2−, 6 = Indistinct (IND). (**C**) Parallel plots for each of the six clusters showing the correlation for the samples within the cluster to a given TNBC subtyping call/method (bottom). Cluster coloring matches that in panel 2B. (**D**) Sankey plot showing how samples reassign from the original TPM call, to the second most correlated call (for re-assignment of IM, MSL, and UNS samples) and their cluster assignment from panel B. (**E**) TNBC clusters (from panel B) and their distribution among SRR. (**F**) Total abundance of tumor-associated leukocytes estimated using CIBERSORT deconvolution methods is shown in comparison to SRR and treatment groups.

A major caveat to treating TNBC is inherent to its clinical diagnosis being lack of hormone receptor expression, which indicates that the most effective hormone-targeted therapy would simply not be beneficial. An effort to determine indications that are actionable includes characterization of genomic expression signatures, which separate TNBC tumors into molecular subtypes [41]. TNBC subtypes were initially determined using the Vanderbilt TNBC subtype tool, which functions by correlating genomic input with pre-determined gene signatures that were discovered in the tool’s tumor training [42]. The original six Vandy subtypes included mesenchymal (M), immunomodulatory (IM), luminal androgen receptor positive (LAR), basal-like 1 (BL1), basal-like 2 (BL2), and mesenchymal stem-cell like (MSL) categories. While two of these categories (IM and MSL) have been retired from use [43], the tool still designates all six categories, and users are advised to manually re-assign these based on the second-highest correlated subtype. We first used the suggested manual reassignment strategy (Figure 2A) and compared subtype distributions between SRR groups. We observed that AAs had the highest proportions of M and BL1, whereas EAs had primarily BL2 (Figure 2A). 

We noted that Vandy categories are inherently heterogeneous, as the tumors show positive correlation with multiple subtypes. Therefore, we created a more data-driven approach using a method, named the Triple Negative Hetero-Fluid (TNHF) method (described in Methods), which measures correlation scores (CS) from only the valid TNBC subtype categories, BL1, BL2, M, and LAR and incorporates the heterogeneity of these subtypes into specific categories. Specifically, we measured the relatedness of our TNHF subtype CS with Vandy TNBC CS, using unsupervised clustering (Figure 2B). Based on the CS patterns, the tumors separated into six cluster nodes, as opposed to the four manually reassigned categories. The CS patterns of each node revealed heterogenic tumor phenotypes, observed as a positive or negative CS for a given category, indicating the presence or absence of the genomic signatures underlying the TNBC subtypes in each tumor (Figure 2C). Therefore, we used a subtype nomenclature to indicate a positive or negative subtype status to describe the heterogeneity. These TNHF category nodes were designated as: 1. LAR+/BL1−, 2. M−, 3. M+, 4. BL2+/BL1−, 5. BL1+/BL2−, and 6. Indistinct (IND—not negatively/positively correlated with any specific subtype) (Figure S4B). These designations allowed us to stay within the framework of the original TNBC subtypes yet capture the breadth of heterogeneity within the naturally occurring phenotypes. Subsequent comparisons of subtypes among the SRR categories now indicated that AAs had the highest occurrence of BL2+/BL1−, and EAs had the highest occurrence of M−. This change in SRR distributions suggests that manual reassignment of categories from the Vandy tool is likely not the optimal resolution. As we tracked how the subtype designations changed from the original tool, through reassignment, ending with the TNHF category, (Figure 2D), we see that manual reassignments led to a collapse of options that mis-categorized certain tumors, that the TNFH approach was able to resolve into genomic data-derived heterogenic subtypes. For instance, Vandy-designated IM tumors were split between LAR and BL2 manual reassignments; however, they were reconnected into the M− TNHF category, which represents multiple negative correlations (Figure S4B), and a lack of homogeneity to fit any Vanderbilt categories. Further investigation of the expression signatures in the M− tumor subtypes, perhaps in a single-cell fashion, is necessary to determine the true biological definition of this tumor subtype.

### 2.4. Differences in Immune Responses by RNAseq Deconvolution

To investigate immunological differences in our AA-enriched TNBC cohort, we used CIBERSORT [44], an in silico deconvolution method, to determine the estimated prevalence of specific immune cell types across the TNBC tumors. We compared proportions of tumor-associated leukocytes (TAL) across patient strata (SRR, ancestry) (Figure 2E and Figure S5), treatment status (treatment naïve vs residual) (Figure 2E), and our six TNBC subtype clusters (Figure S5). There were distinctions of immune responses, defined by the absolute TAL score (Figure S5A). When comparing high vs low overall TAL scores (adjusted *p*-value < 0.05), 832 genes showed significant differential expression, of which 116 genes are included in the TAL algorithm. The remaining genes associated with TAL scores have contrasting expression across our cohort, indicating significant variation of these immune-related genes between high vs low TAL groups (Figure S5A). Individual TAL scores indicate variation of specific immune cell infiltration; however, the low numbers of individuals in each patient strata precluded our ability to find statistically significant differences, though there are clear trends of higher TAL scores for EAs compared to AAs and lower TAL scores for in pre-treatment vs post-treatment (residual) tumors (Figure 2F).

### 2.5. Potential Druggable Targets from Ancestry-Associated Gene Signatures

**Figure 3:**
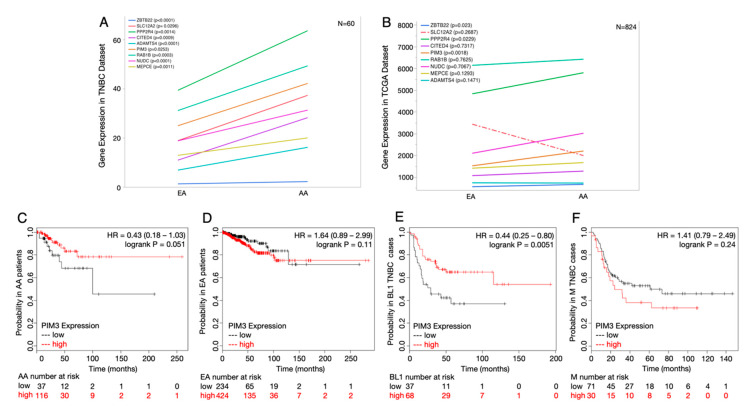
African ancestry-associated genes that are current drug targets in cancer treatments show different survival outcomes between SRR groups and breast cancer subtypes. (**A**) Gene expression levels between QGA differentially expressed genes identified as drug targets between SRR CAs and AAs in our TNBC dataset. (**B**) Gene expression levels between QGA differentially expressed genes in Figure 3A, but from the TCGA cohort. (**C**) Relapse-free survival curve of *PIM3* for SRR AAs shows that higher expression of *PIM3* is associated with higher probability of relapse-free survival (*p* = 0.051). (**D**) Relapse-free survival curve for *PIM3* for SRR CAs shows that higher expression of *PIM3* is associated with lower probability of relapse-free survival (*p* = 0.11). (**E**) Relapse-free survival curve of *PIM3* for TNBC basal-like 1 tumors shows that higher expression of *PIM3* is associated with higher probability of relapse-free survival (*p* = 0.0051). (**F**) Relapse-free survival curve of *PIM3* for TNBC mesenchymal tumors shows that higher expression of *PIM3* is associated with lower probability of relapse-free survival (*p* = 0.24).

As a clinical follow-up to our ancestry-associated gene expression differences, including key biological distinctions in canonical cancer-related networks across both treatment-naïve and treated TNBC tumors, we investigated if these genes are potential drug/treatment targets. Specifically, we performed a search of the literature and www.clinicaltrials.gov to determine if the African ancestry-associated genes we identified have FDA-targeted therapeutics available or are being studied in clinical trials for use in any cancer type. We found more than a dozen African ancestry genes that are currently targeted with FDA-approved drugs, and most of these genes had multiple drug options (Table 1). To determine if these genes are indeed targets for African American TNBC patients, we first verified that they were differentially expressed between SRR groups (Figure 3A) and found they were all significantly different between AA and EA patients. Next, we utilized the TCGA cohort to validate, independently, if the TNBC-specific expression differences were replicated in an additional cohort of patients (Figure 3B). Most of the candidate target genes maintained the same trend; however, statistical significance was lost for most genes, likely due to the lack of ancestry estimation in TCGA tumors, thus the confounding impact of mixed ancestry because of possible discordance between SRR and QGA. However, from our cohort, *PIM3*, *PPP2R4*, and *ZBTB22* retained significant upregulation in the TCGA data (*p* = 0.0018, 0.0229, and 0.0230, respectively) (Figure 3B), suggesting that they have the most robust association with African ancestry that transcends admixture in races. We further investigated the clinical association of the most significant candidate gene, *PIM3*, by evaluating its association with survival, race, and subtype (Figure 3C–F). Higher expression of *PIM3* was protective for AAs and in the basal-like 1 subtype of TNBC. This paradox in expression vs. survival for *PIM3* underscores the need for additional biological validations and contextual information to determine genetic ancestry in addition to molecular TNBC subtyping.

## 3. Discussion

TNBC, the most aggressive form of breast cancer, has limited treatment options. It is characterized by poor overall survival, with recurrent, distant metastatic disease common within the first three years after aggressive chemotherapy treatment. TNBC disproportionally affects young AA women, and there is increasing evidence that this disparity cannot be attributed to solely to SES and lack of access to care. Our previous studies [14,45,46] and others [47] have demonstrated differences in gene expression based upon race. However, since SRR does not allow more than correlation with African ancestry, Quantified Genetic Ancestry (QGA) analyses are needed to understand the shared genetic drivers of TNBC observed across the modern African Diaspora [6,48]. Furthermore, due to the heterogenicity of TNBC, additional tools are required to define TNBC subtypes and ancestry-related differences within TNBC subtypes. Herein, we used two newly developed tools to evaluate the heterogeneity of TNBC for patients with African ancestry. 

Prior studies that compare SRR groups for differential gene expression in breast and other cancers have revealed significant differences between AA and EA race groups [14,45,46,47]; however, many of these changes are confounded by genetic admixture and non-genetic factors that prevent clear interpretation of genetic contributions to SRR differences in tumor biology. By use of QGA, we identified ancestry-related differential expression of genes in treatment-naïve and residual TNBC tumors which were involved in canonical cancer pathways, but had predictions of modified functional activity. This deconvolution of ancestry has also been employed in prostate cancers, also revealing gene expression correlated with specific West African ancestry [49]. We observed that specific pseudogenes, that showed reduced levels of expression associated with African ancestry, are located in regions of the genome that are frequently deleted in sporadic breast and prostate tumors derived from AA patients [50]. An example of this expression/deletion effect involves a pseudogene, *RNU2-6p*, which is downregulated in AA TNBCs (Figure S6). According to GTEx data, this gene is not typically expressed in normal breast tissue; however, it is highly expressed in breast tumors within our cohort, but with significantly reduced expression in untreated TNBC tumor of patients with significant African ancestry. The functional relevance of this distinction, based on in silico analyses and previously published findings [51,52], is that *RNU2-6p* appears to be a non-coding nuclear RNA that has a secondary structure resembling splicing machinery. This ancestry-associated pseudogene may affect exon usage and/or isoform splicing, which may contribute to unique molecular signatures in gene expression, translating into disease progression in African Americans with breast cancer, as has been previously shown in prostate cancer [53,54].

When we compared the differential genes identified by QGA with those identified by SRR, we found a 51% overlap of ancestry-associated genes in the race-associated category. This indicates that using SRR categories for differential gene expression can diminish ancestry-related expression, given the convolution of admixture in race groups, and SRR categories will incorporate additional factors that drive differences in gene expression that are independent of genetic ancestry. This also explains the relatively larger number of differentially expressed genes associated with SRR, as opposed to genetic ancestry, and provides additional opportunities to discern the multiple factors connected to race/ethnicity that contribute to differential gene expression among race groups.

Additionally, despite the limited number of residual tumors in our cohort, we also observed a robust 13-gene expression pattern upregulated in AAs but not in EAs with residual tumors. Of note, EGFR, which is upregulated in African American breast and prostate cancers [55,56], appears to be a driver within this gene signature. Additionally, genes that are downregulated in AAs had a strong expression in EA patients. These differences did not correlate with TNBC subtypes as determined by use of either the Vanderbilt or TNHF subtyping tools, suggesting that these genes are likely due to genetic ancestry. Furthermore, in the TNHF analysis, there were fewer unclassified AA patients. This has prognostic implications, since, for TNBCs, residual disease after neoadjuvant chemotherapy is associated with worse overall survival relative to that for non-TNBC patients, which is not the situation when patients achieve a complete pathologic response [57,58,59]. Thus, identification of genes that are drivers in residual tumors can help in developing targeted adjuvant therapies that could improve survival in this patient population, for which there currently exists no effective standard of care.

The TNBC Vandy BL1/2 distribution between SRR groups was different from our previous findings in TCGA analyses [14], likely because of inclusion all six TNBC subtype categories in that previous study. Specifically, reassignment of IM- and MSL-retired subtypes calls resulted in redistribution of tumors into sub-optimal categories, shifting the observed proportions of subtypes in SRR from our previous studies (Figure 2A). This contradiction compelled us to ensure that the categorization of subtypes was an accurate interpretation of the biological variation across TNBC tumors, and not just a reflection of an improperly stratified training set. Our pilot utilization of the novel TNHF method, an augmented extension of the Vanderbilt tool, is distinctive in various ways. First, TNHF reports only the correlation scores for valid TNBC categories. Second, TNBC categories from TNHF are assigned as a semi-quantified ‘status’, which represents the presence/absence of a mixture of valid Vanderbilt TNBC subtypes within tumors, which corresponds to heterogeneity observed in TNBC tumors. Because this TNHF method allows us to account for subtype heterogeneity within a tumor—denoted as positive or negative annotations—this dynamic output allows for a comprehensive account of proportions of TNBC subtype signatures that may be more informative for clinical management of breast tumors. This can be transformative in TNBC disease outcomes, as certain TNBC subtypes exhibit a higher risk of recurrence and/or drug resistance. Therefore, information of mixed tumor types may help predict adverse outcomes or limited treatment response and tumor evolution in the context of residual tumor behavior. In our cohort, African ancestry patients had a higher rate of basal-like 2 positive/basal-like 1 negative (BL2+/BL1−) TNBC subtypes, which is similar to previous findings for AA patients [14,47]. This positive/negative integration of all potential TNBC categories, which have prognostic value, has added clinical utility, particularly for making treatment decisions. The capacity of gene expression profiles to predict treatment response is supported by clinical trial data showing differences in pathological complete responses based upon Vanderbilt TNBC subtypes [60,61,62]. For example, in the GEICAM/2006-03 TNBC neoadjuvant chemotherapy clinical trial, the best responders were in the BL1 group, with 60% of patients achieving a pathologic complete response compared to 20% in the LAR and IM groups [60]. Thus, use of the more refined TNHF subtyping tool, which can provide information such that a tumor is equally BL2+ and M+, can have a greater impact on neoadjuvant treatment decisions and can inform subsequent choices if standard treatments fail.

Both BL2/BL1 subtypes are also associated with immune gene signatures for AA TNBC patients [14,63,64], which appears to be driven by IL-6 and TP53 signaling as determined by IPA. Both IL-6 [65] and p53 activation [66,67,68,69,70] are associated with African American tumors, validating the robustness of our analysis tools. Although we found no significant associations with Tumor Associated Leukocyte (TAL) scores, most likely due to samples being isolated from macro-dissected regions enriched for tumor cells and depleted of stromal and/or highly infiltrated regions, tumors of patients with significant African ancestry corresponded with lower TAL score compared to patients with predominantly European ancestry among treatment-naïve patients. In the tumor microenvironment, various genes, including immunological genes, are differentially expressed by race/ethnicity [46,71]. However, some studies that utilize public datasets that have low representation of ethnic groups indicate that immunological differences in TNBCs are relatively small [72]. Although, at the individual level, we found a difference in lymphocytic infiltration, it was not obvious at the race/ethic group level, may be due to small sample numbers in each race/ethnic group. However, higher TAL scores for EAs and lower TAL scores for treated, residual tumors were noted. A TNBC study of south Asian patients has reported increased infiltration of T-lymphocytes [73] and suggest that TNBCs with higher immunogenicity may be candidates for immunotherapy [74]. Thus, higher TAL scores observed in our EA TNBC patient cohorts could be exploited to select the relevant immunotherapies.

## 4. Materials and Methods 

### 4.1. TNBC Patient Cohort and Sample Collection

To identify molecular signatures that differ between TNBCs of AA women and EA women, we performed RNA sequencing (RNAseq) on a TNBC cohort. A retrospective convenient formalin-fixed, paraffin-embedded (FFPE) archival tissue cohort from the Division of Anatomic Pathology of University of Alabama at Birmingham (UAB) consisting of 104 AA and EA women diagnosed with TNBC between 2000 and 2012 was selected for this study (Figure S1A). All samples were collected and utilized in this study with the prior approval of the UAB Institutional Review Board (IRB number: 060911009). Personal medical history and clinical records were limited for this cohort. Following quality control screening, a final set of 75 cases remained (42 AAs and 33 EAs). Of these, samples were separated by treatment status treatment-naïve (*n* = 60) or residual tumors (*n* = 15). Of the treatment-naïve cases, there was a near equal distribution of race categories (31 AAs and 29 EAs). Of the residual tumor cases, the representation of AAs was more than twice that of EA (11 AAs and four EAs). All tumors and corresponding normal regions were macro-dissected by pathologists prior to RNA extraction. Stage and grade distribution were similar between race groups (Table S3). 

### 4.2. RNA Extraction, Library Preparation, and Primary Analysis

RNA was extracted from macro-dissected samples using standard RNA extraction kits. The concentration and integrity of the RNA was estimated by a Qubit® 2.0 Fluorometer (Invitrogen, Carlsbad, CA, USA) and an Agilent 2100 Bioanalyzer (Applied Biosystems, Carlsbad, CA, USA), respectively. Total RNA from each sample was taken into RNAseq applications. First, ribosomal RNA (rRNA) was removed with Ribo-Zero™ Gold kits (Epicenter, Madison, WI, USA) by the manufacturer’s recommended protocol. Then, the RNA was fragmented and primed for the first-strand synthesis using the NEBnext First Strand synthesis module (New England BioLabs Inc., Ipswich, MA, USA). Second-strand synthesis was then performed with the NEBnext Second Strand synthesis module. Following this, the samples were taken into a standard library preparation protocol using NEBNext® DNA Library Prep MasterMix Set for Illumina® with slight modifications. Briefly, end-repair was accomplished, followed by A-tailing and custom adapter ligation. Post-ligated materials were individually barcoded with unique in-house Genomics Service Laboratory (GSL) primers. Library quantity was assessed with a Qubit 2.0 Fluorometer, and the library quality was estimated by utilizing a DNA 1000 chip on an Agilent 2100 Bioanalyzer. Quantification of the final libraries for sequencing applications was determined using qPCR-based KAPA Biosystems Library Quantification kits (Kapa Biosystems, Inc., Woburn, MA). Paired-end sequencing was performed with an Illumina HiSeq2500 sequencer (Illumina, Inc., San Diego, CA, USA).

### 4.3. Quality Control and Sequence Alignment

Fast QC (version 0.11.8) was used to perform quality control on the raw sequencing reads [75]. To proceed through the analysis with high-quality reads, adapters and low-quality sequences were trimmed from the raw reads using Trimmomatic (version 0.36) [76]. These reads were then aligned to the reference genome (GRCh37 assembly) using HISAT2 (version 2.0.4) [77]. Although rRNA reduction steps were taken during library preparation, we removed any remaining rRNA contamination in the samples using the Bed-tools (version 2.26.0) intersect function against a bed file of annotated rRNA sequences [78]. Following quality assessment of sequence data, 28 cases were excluded due to sequencing artifacts (Figure S1B). 

### 4.4. Gene Expression Quantification and Differential Gene Expression Analyses

After alignment and rRNA gene reads removal, RNAseq alignments were assembled into potential transcripts, and gene expression levels were quantified using Stringtie (version 1.3.3) [77]. All comparative analyses for differential gene expression were conducted within the respective treatment groups, treatment-naïve or residual tumors. To identify genes that were ancestry-associated, we used JMP® Version 14.0 (SAS Institute Inc. Cary, NC, USA) to conduct a gene-by-gene linear regression model, testing the quantified (continuous) measurements of African and/or European genetic ancestry against the gene expression levels. False-discovery Rate (FDR) adjusted p-values were used to determine significant associations. DESeq2 was then used to validate whether genetic ancestry-associated genes were differentially expressed between self-reported AA and EA individuals [79]. Fold-change values from both the ancestry-associated gene lists and SRR gene lists were used in IPA (see below).

### 4.5. TNHF TNBC Subtyping

To determine the prevalence of TNBC subtypes in the cohort, we first utilized the *Vanderbilt TNBC subtyping* tool to identify basal-like 1 and 2 (BL1 and BL2), immunomodulary (IM), luminal androgen receptor (LAR), mesenchymal (M), and mesenchymal stem-like (MSL) tumor samples [41,42]. These six subtypes were further refined to four TNBC subtypes, with re-assignment of IM and MSL subtypes, as these are primarily composed of immune and stromal cell populations, respectively [43]. To address this in our variant calls from the Vanderbilt TNBC type tool, samples that were assigned IM or MSL were re-assigned to their second most correlated TNBC subtype. As a supplementary validation method to the Vanderbilt TNBC classification tool, a summarized ranks measure was computed using the original TNBC subtype signatures for all samples using normalized RNAseq expression data. TNBC subtype signatures were obtained from Lehmann et al. [41]. Across all samples, genes were ranked from low to high expression using the rank function in R statistical software with the minimum rank method used to resolve duplicate expression ties. For each sample, ranks for each gene in the given subtype signature were extracted, and a representative median or mean of ranks for the gene signature was calculated to estimate the overall regulation of the signature with respect to the total expression. The TNBC subtype signature with max median or mean signature rank per sample was the assigned TNBC subtype for the sample. Where max median or mean rank was used, it is denoted in figures as TNHF-Median or -Mean, respectively.

### 4.6. Genetic Ancestry and Admixture Estimations from RNAseq Single Nucleotide Variants (SNV)

Genetic ancestry was determined using *Admixture* (version 1.3.0) [80], which provides a maximum likelihood estimation of individual ancestries from multi-locus SNVs. Prior to admixture analysis, GATK best practices were used to identify SNVs from the RNAseq reads. Specifically, we aligned our RNAseq reads to hg19 using STAR (version 2.5.2b) [81]. Variants were called using GATK HaplotypeCaller (version 3.8) [82,83] and subsequently filtered to exclude rare variants (i.e., <5% across all phase 3,1000 genomes), all INDELs, and any SNPs that were not biallelic. Ancestral reference populations were based on the 1000 Genomes Project phase 3 superpopulations [84].

### 4.7. Gene Network Analyses

To complete in silico analysis of predicted gene interactions and enrichment of functional pathways, we utilized IPA software (version 01-16) (QIAGEN Inc., https://www.qiagenbioinformatics.com) to analyze the ancestry-associated and SRR differentially expressed gene lists [40]. After uploading each respective dataset, we filtered out any differentially expressed gene that was not significant at a threshold of *p* < 0.05. In the core analysis, IPA takes in the differential expression data and uses the log-fold expression change values in coordination with the curated Ingenuity Knowledge Base to identify top signaling and metabolic pathways, upstream regulator molecules, and associations with various diseases and bio-functions. Significance of networks was based on the score (where a score of ≥ 3 indicates with > 99% confidence that the network was not generated by random chance).

### 4.8. Estimation of Tumor-Associated Leukocyte Populations

The CIBERSORT [44] online platform was used to determine the estimated abundance of tumor-associated immune cells in our tumor samples. The analysis was completed with 500 permutations, and quantile normalization was disabled, as recommended for RNAseq data input. The absolute score for a given tumor-associated leukocyte (TAL) population represents the estimated abundance of immune cell types in the tumor sample. The CIBERSORT absolute score represents the abundance of all 22 tumor-associated leukocyte (TAL) populations scored as identified by the tool. TAL absolute scores were dichotomized into low and high categories using lower-quantile distribution.

### 4.9. Survival Analyses

Kaplan–Meier Plotter (KMPLOT) [85,86] was used to determine gene expression-associated survival outcomes. Our analysis was based on the version of data available (last accessed 12/2019). To determine the association between survival outcomes and *PIM3* gene expression within SRR race groups, we used the KMPLOT TCGA pan-cancer breast cohort platform [86]. Relapse-free survival curves were calculated comparing low versus high *PIM3* expression among AA (*n* = 153) and EA (*n* = 658) groups. To determine association of survival outcomes and *PIM3* gene expression among TNBC subtype groups, we used the Kaplan–Meier Plotter breast cancer platform, which included TNBC cases from a collection of 35 GEO datasets provided through the KMPlot.com analysis platform [85]. Relapse-free survival curves comparing low versus high PIM3 expression were assessed among BL1 (*n* = 105) and M (*n* = 101). Hazard ratios and *p*-values are reported in the figure panels for each survival analysis. The auto-select best cutoff option was used to determine the cut-off for high versus low *PIM3* expression within each analysis group in both tools. For each analysis, this considers all cutoff values between the lower and upper quartiles for *PIM3* expression, and selects the best performing value to distinguish high verses low expression. Numbers of *PIM3* high- versus low-expressing individuals at each time interval (in months) is shown as survival tables within each KM plot.

## 5. Conclusions

Genes that exhibit ancestry-specific regulation, particularly those with cancer-related function, are a valuable resource from our findings. Pointedly, targeted therapeutics that are presently undergoing clinical trials or have received FDA approval match several of our ancestry-associated genes that were differentially expressed in our cohort. Further validation of these genes’ differential expression between race groups in the Breast Cancer TCGA RNAseq cohort demonstrates the reliability of our findings and the likelihood of translational impact. Of note, in both cohorts we found higher levels of *PIM3* and *PPP2RA* in TNBC tumors from African-American patients. However, in independent validation cohorts from GEO and TCGA, survival analyses indicated that high expression correlated with divergent clinical outcomes between race groups, as high *PIM3* expression correlated with better survival for patients of African ancestry, compared with a worse overall survival for the EA patients. Limitations to stratify public data with correlated demographic data and missing survival data in our cohort preclude definitive actionable clinical conclusions at this time. Although there are ongoing efforts to recruit minorities into existing clinical trials [87,88], these findings highlight the impactful possibilities of utilizing genetic ancestry in multi-ethnic cohorts and the need to demonstrate that variation within and among TNBC subtypes and how genetic ancestry may impact tumor biology, which in could guide treatment decisions.

## Figures and Tables

**Table 1:** Genes involved in networks with African ancestry-associated genes are potential therapeutic/disease management targets with currently utilized treatments.

Gene	Name	Drugs Tested in Cancer	Disease (Cancer or Other)	Organism	Pubmed ID (PMID)
*AKT1*	AKT Serine/Threonine Kinase 1	Arsenic Trioxide, Carboplatin, Everolimus, Cisplatin, Nelfinavir	Various Cancers	Human	12480548
*CCND1*	Cyclin D1	Arsenic Trioxide, Cetuximab, Aspirin, Trametinib, Palbociclib	Various Cancers and other diseases	Human	12480548
*SLC12A2*	Solute Carrier Family 12 Member 2	Bumetanide and Furosemide	Neonatal Seizures, Autism, Heart Failure	Human	11698253
*PPP2R4*	Protein Phosphatase 2 Phosphatase Activator	Ceramide	Breast Cancer, Diabetes, Obesity	Human	29261144
*RELA*	RELA Proto-Oncogene, NF-KB Subunit	Dimethyl fumarate	Multiple Sclerosis	Human	26683377
*CITED4*	Cbp/P300 Interacting Transactivator With Glu/Asp Rich Carboxy-Terminal Domain 4	Fluorouracil	Cardiac ischaemia/reperfusion (I/R) injury	Mouse	28304151
*PIM3*	Pim-3 Proto-Oncogene, Serine/Threonine Kinase	Fostamatinib, Gefitinib, Sunitinib, Ruboxistaurin	Cancer and others	Human	26516587
*EGFR*	Epidermal Growth Factor Receptor	Gefitinib, Erlotinib, Lapatinib and Cetuximab	Non-small cell lung cancer	Human	15118073
*LPL*	Lipoprotein Lipase	Orlistat, Fenofibrate	Obesity and Diabetes	Human	24016212
*NUDC*	Nuclear Distribution C, Dynein Complex Regulator	Phenethyl Isothiocyanate	Various Cancers and Cardiovascular Disease	Human	21838287
*MEPCE*	Methylphosphate Capping Enzyme	S-Adenosyl methionine	Various	Human	23985780
*IL6*	Interleukin 6	Siltuximab, Vitamin C and E, Adalimumab	Various	Human	8823310
*NFKB1*	Nuclear Factor Kappa B Subunit 1	Thalidomide, Donepezil, Glycyrrhizin, Triflusal	Various	Human	15723633
*ADAMTS4*	ADAM Metallopeptidase with Thrombospondin Type 1 Motif 4	Tinzaparin	Brain Tumors, Thromboembolism, Thrombosis	Human	15723278
*TP53*	Tumor Protein P53	Venetoclax, Cyclophosphamide, Fluorouracil, Cisplatin	Various	Human	27069256

## Supplementary Materials

The following are available online at https://www.mdpi.com/2072-6694/12/5/1220/s1. Figure S1: Cohort sample description and primary quality control (QC), Figure S2: Differential gene expression analysis of treatment-naïve tumors using self-reported race (SRR), Figure S3: Differential gene expression analysis of residual, post-treatment tumors using SRR reveals 13 race-specific genes distinct from treatment-naïve tumors, Figure S4: TNBC subtyping methods and distribution, Figure S5: CIBERSORT deconvolution of TNBC tumor samples, Figure S6: RNU2-6P is significantly downregulated in AA tumors compared to EA, but is not typically expressed in normal breast tissue, Table S1: Top differentially expressed genes from SRR treatment-naïve analysis, Table S2: Top differentially expressed genes from SRR residual tumor analysis, Table S3: Cohort Clinical Attributes, Table S4: Significantly differentially expressed genes associated with quantified genetic ancestry among treatment-naive tumors, Table S5: Significantly differentially expressed genes associated with self-reported race among treatment-naive tumors.
