# Supplementary material for: Identification of Distinct Heterogenic Subtypes and Molecular Signatures Associated with African Ancestry in Triple Negative Breast Cancer Using Quantified Genetic Ancestry Models in Admixed Race Populations
Source: Cancers (Basel). 2020 May 13;12(5):1220. doi: 10.3390/cancers12051220 (PMC7281131; doi:10.3390/cancers12051220)
Supplement: Supplementary file 1 [file cancers-12-01220-s001.zip › cancers-785734 supplementary/Supplementary2020-04-29/SUPPLEMENTAL FIGURES AND TABLES FOR SUBMISSION.docx]

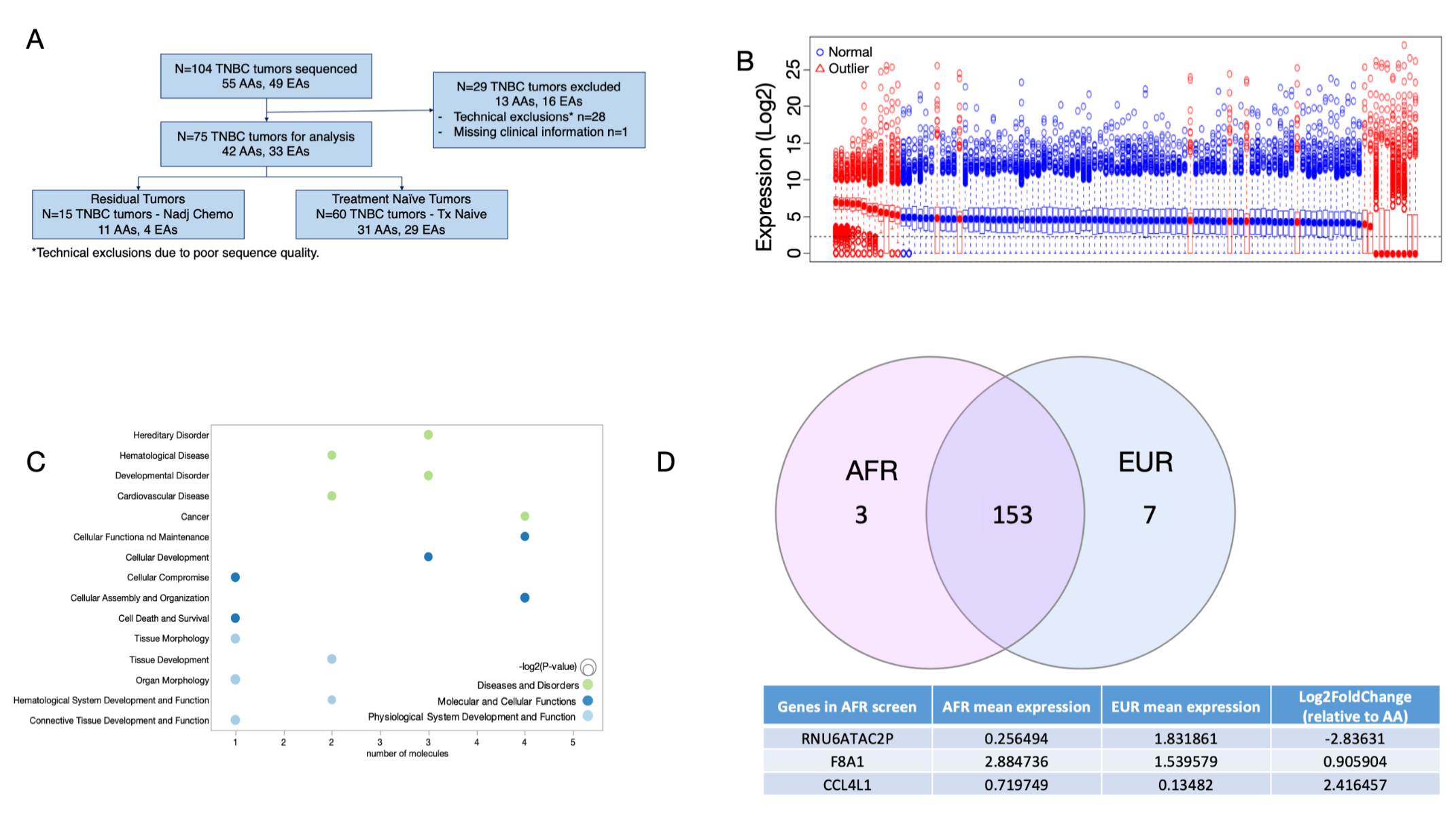


**Figure S1.** Cohort sample description and primary quality control (QC). (**A**) Study design of the bi-racial cohort, including loss of samples to technical exclusions related to sequence quality (*n* = 28) and missing clinical information (*n* = 1). (**B**) QC assessment of the relational behavior of all sequenced samples prior to exclusion of technically poor samples (left, denoted as red triangles), and after their exclusion (right).


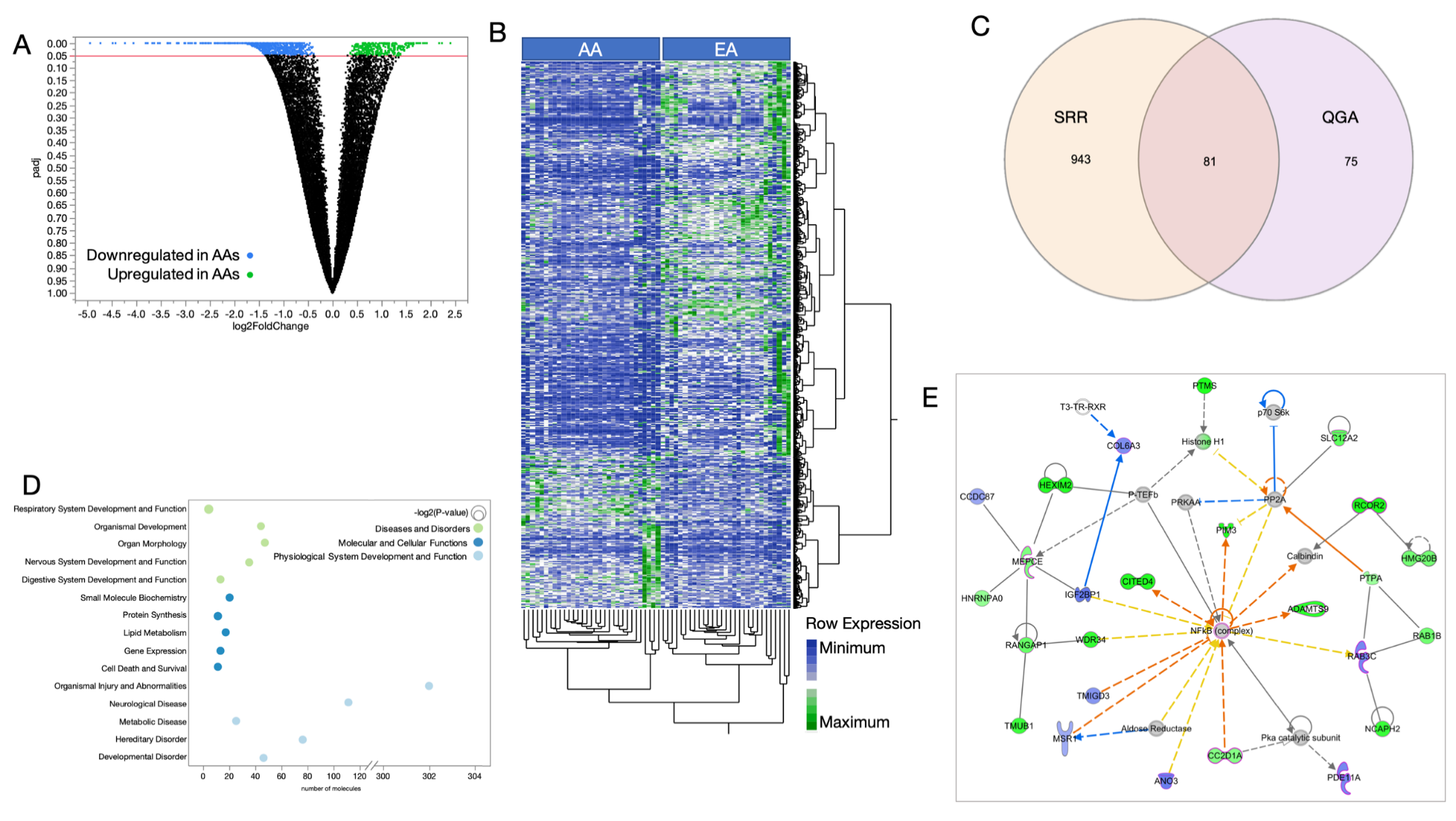


**Figure S2. Differential gene expression analysis of treatment-naïve tumors using self-reported race (SRR).** (**A**) Volcano plot showing genes differentially expressed by SRR. A p-value threshold of P < 0.05 is indicated by the red line, where the green dots represent 297 genes upregulated in AAs, and blue dots indicate the 764 genes downregulated in AAs. (**B**) Venn diagram showing overlap of DEGs from SRR race analysis versus QGA analysis. (**C**) Clustergram heatmap of the 1061 genes (*p* < 0.05) that show differential expression between SRR race groups. Rows represent genes, and columns represent individuals. From the gene expression patterns, individuals separate into distinct AA and EA nodes (hierarchical structure on bottom, race labelled on top). (**D**) Top significant bio-functions and diseases from the SRR DEG analysis from Ingenuity Pathway Analysis. (**E**) Top canonical pathways represented from the SRR DEG analysis. Blue represents proportion of downregulated genes; green represents upregulated; and white represents molecules in the canonical pathway that are not in the differential gene set.


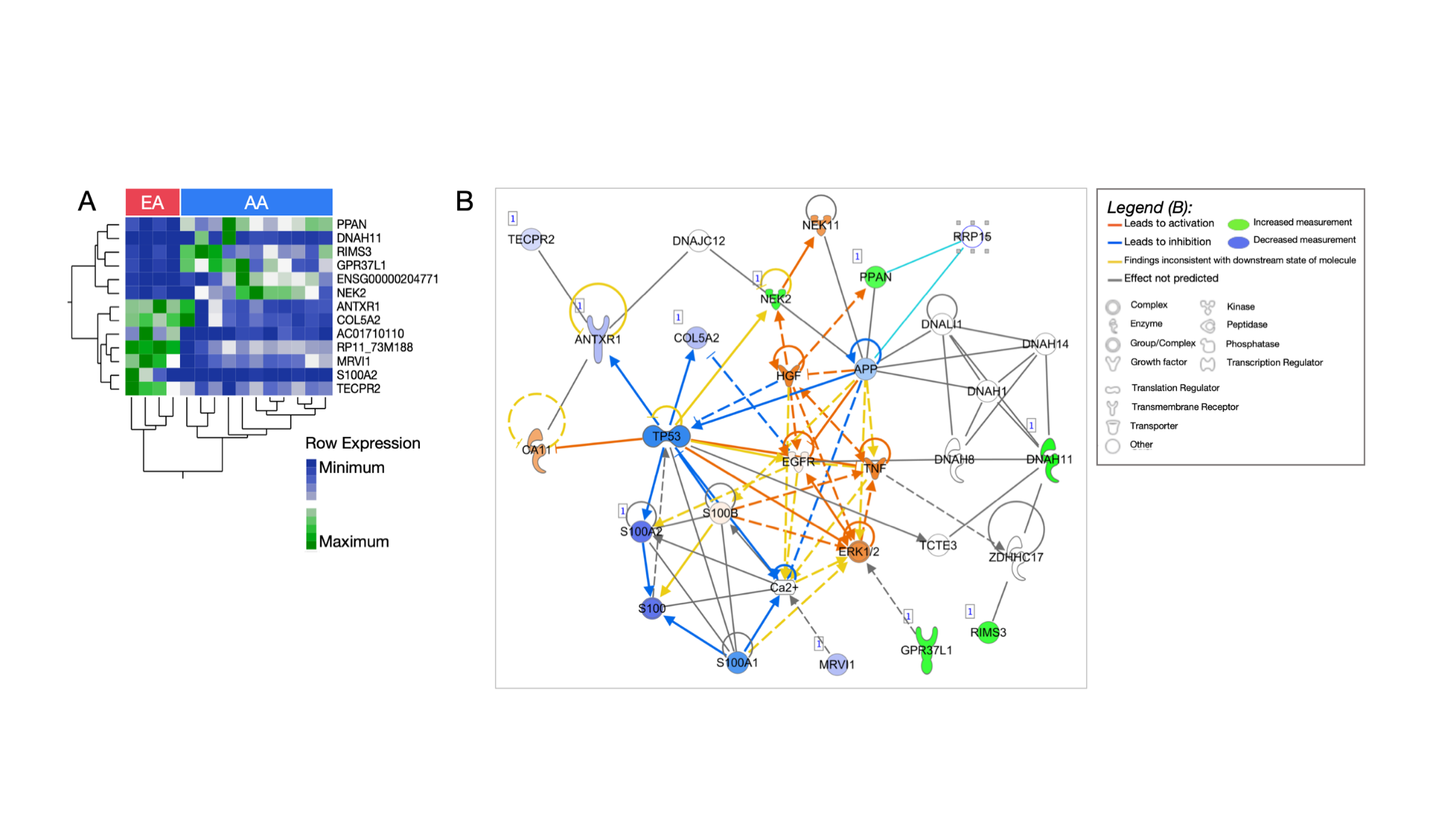


**Figure S3. Differential gene expression analysis of residual, post-treatment tumors using SRR reveals 13 race-specific genes distinct from treatment-naïve tumors.** (**A**) Clustergram heatmap of 13 genes (*p* < 0.05) that were differentially expressed by SRR among residual tumor samples. Rows represent genes (labelled, right), and columns represent individuals. SRR is shown in the top row of the color map (red indicates CA, and blue indicates AA). Minimum row/gene expression is indicated by blue, and high expression is indicated by green. (**B**) *De novo* network analysis using SRR DEGs from residual tumors. Molecules in green are upregulated in AAs, and those in blue are downregulated in AAs. Colors indicating activation and interactions are shown in the key and described in Figure 1D.


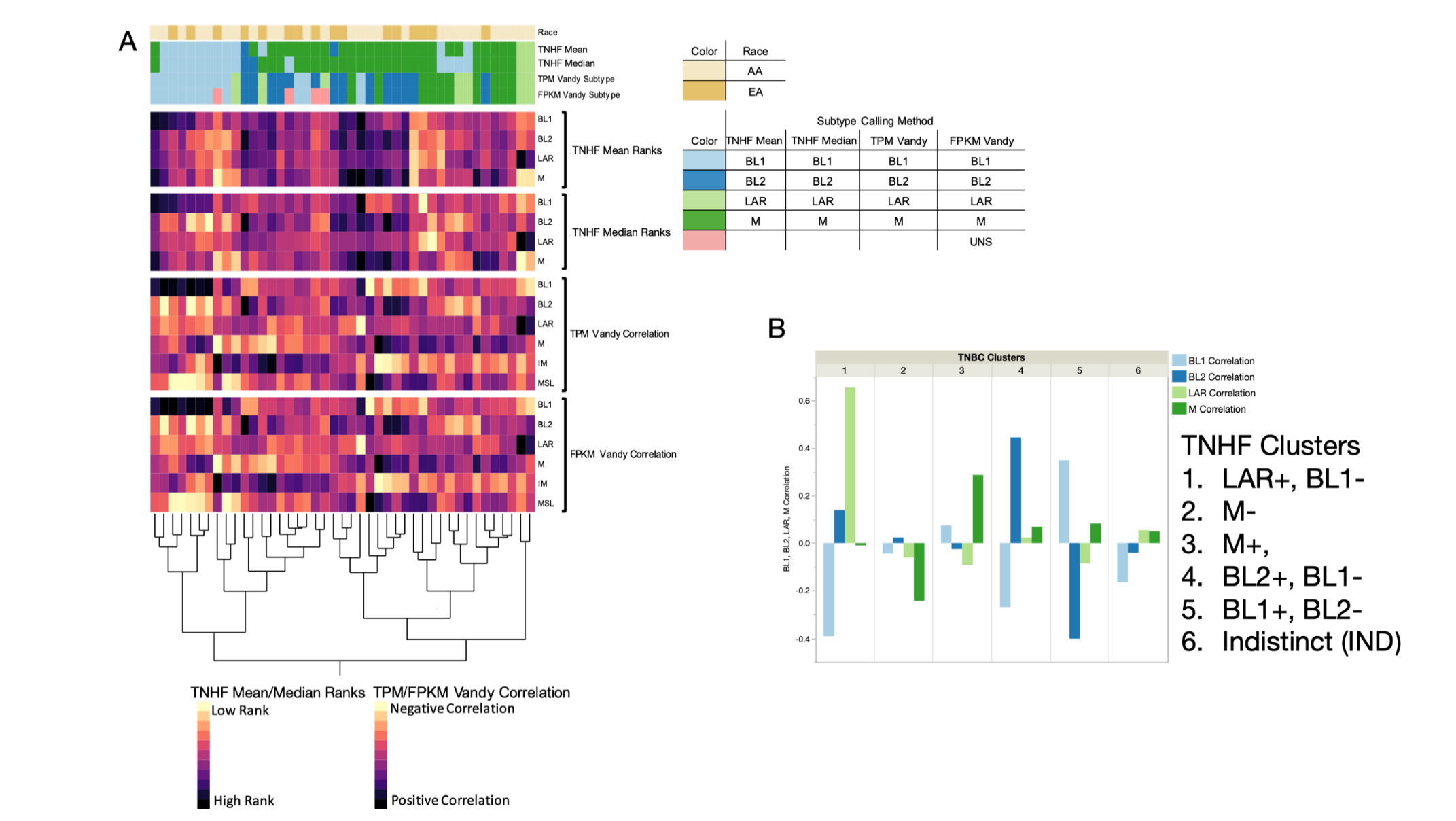


**Figure S4. TNBC subtyping methods and distribution.** (**A**) Clustergram heatmap showing the correlation and rank output from our TNBC subtyping methods in comparison to the Vanderbilt methods, using both FPKM and TPM gene expression values as input. In the top color map, the first row indicates SRR, where SRR AAs are light gold, and EAs are deep gold (SRR key, top right). The next four rows of the color map indicate the consensus TNBC subtype call for the tools used (TNHF Mean ranking calls, TNHF Median ranking calls, Vanderbilt tool with TPM input, Vanderbilt tool with FPKM input). The color key is to the right, where light blue is BL1, dark blue is BL2, light green is LAR, dark green is M, and light pink is UNS calls. The heatmap section is broken into subsections with ranks for the TNHF tool, and subtype call correlations for the Vanderbilt tool, with light yellow/orange indicating low-rank/negative correlation, and dark purple indicating high-rank/positive correlation with a given subtype (right). Hierarchal clustering of the samples (columns) are shown at the bottom. (**B**) Pie charts showing the distribution of TNBC subtypes called by the Vanderbilt method (TPM input) and our calling method (TNHF mean ranking) across the TNBC cluster columns defined in Figure 2B. Colors for TNBC subtype are shown to the right.


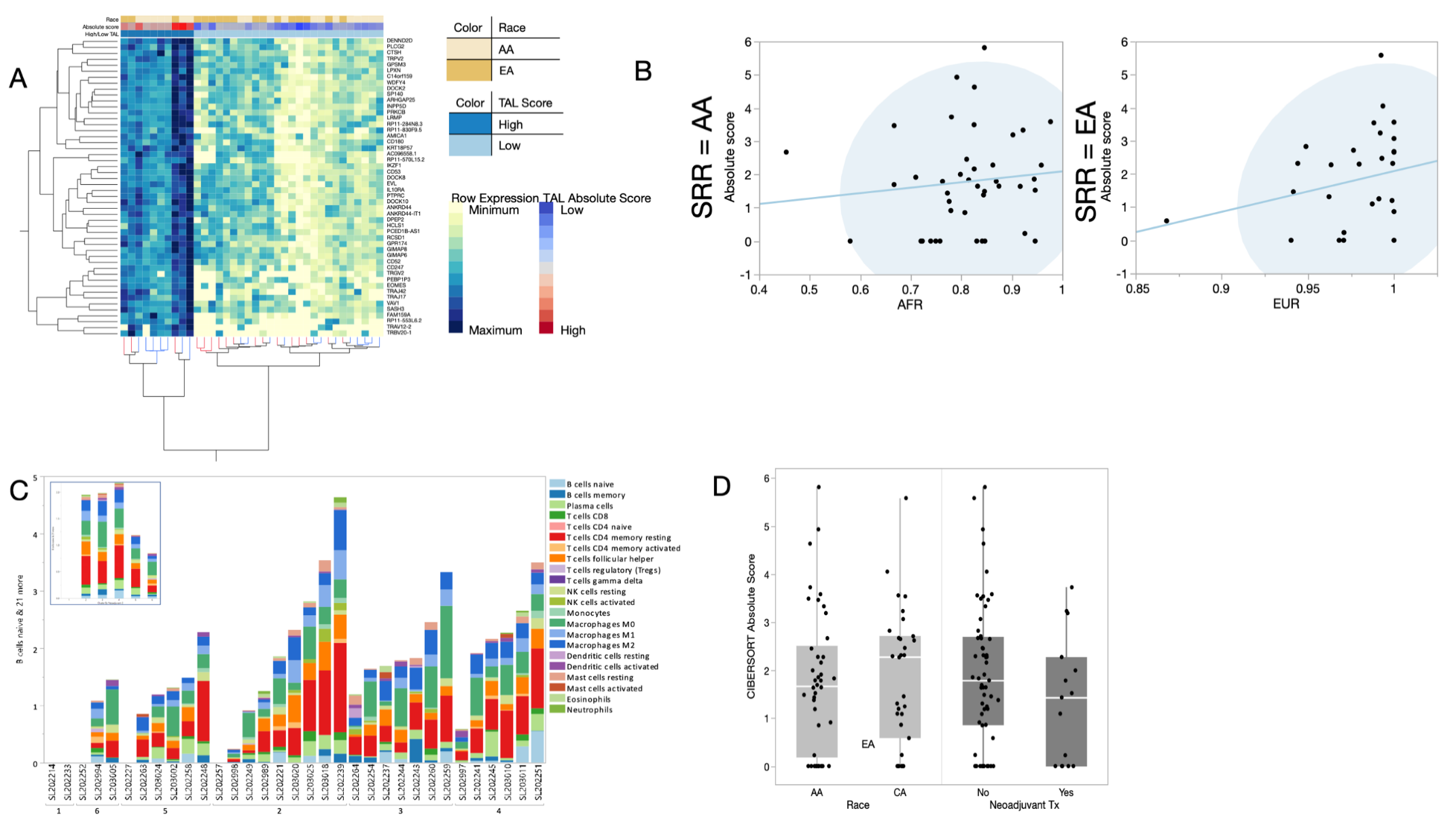


**Figure S5. CIBERSORT deconvolution of TNBC tumor samples.** (**A**) Clustergram heatmap showing the top 50 genes (*p* < 0.0001) differentially expressed between tumors with low or high CIBERSORT tumor-associated leukocyte (TAL) absolute scores. Rows represent genes (labelled, right), and columns represent individuals. Lower gene expression is shown in yellow and higher expression in blue. The color map columns indicate race (light gold, AAs, darker gold, CAs), TAL scores (continuous, blue indicating low scores, red indicating high scores), and High/Low TAL (dichotomized, light blue, low, dark blue, high). Hierarchical structure of genes is shown on the left, and the hierarchical structure for individuals is shown on the bottom. (**B**) CIBERSORT TAL absolute scores among treatment-naïve (neoadjuvant treatment, no) and residual tumor (neoadjuvant treatment, yes). These groups are additionally broken down by SRR, with AAs in light blue and CAs in dark blue. (**C**) CIBERSORT TAL absolute scores by race and neoadjuvant treatment status.


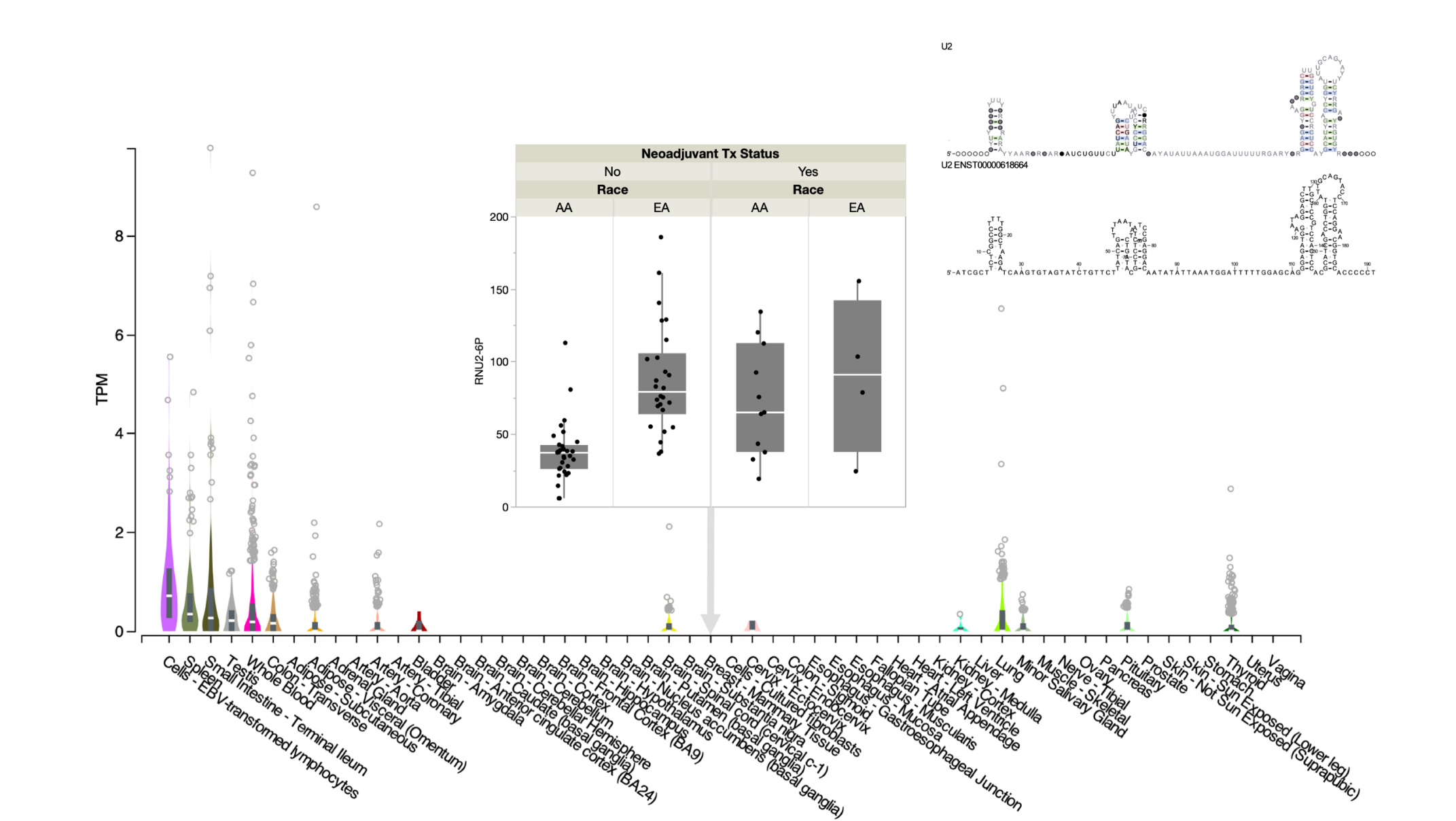


**Figure S6. RNU2-6P is significantly downregulated in AA tumors compared to EA, but is not typically expressed in normal breast tissue.** Expression analysis of a pseudogene, RNU2-6P indicates it is expression in normal tissues (GTEx cohort) including; peripheral blood cells, spleen, intestine, testes, colon, adrenal gland, endothelial tissues, lung and thyroid. Previous studies show that this gene has secondary structure similar to splice regulators. In our study (center box plot inset) we show a significant down regulation in AA treatment naïve cases. Interestingly the gene is not shown to be significantly expressed in normal tissue. Independent studies from our group indicate that the location of the gene overlaps with deleted regions that are conserved deletions among breast and prostate cancer patients of African descent.

**Table S1. Top differentially expressed genes from SRR treatment-naïve analysis.**

|  | **Gene** | **Log_2_Fold Change** | ***p* Value** |
| --- | --- | --- | --- |
| **Upregulated in SRR AA compared to EA** | *OVOS2* | 2.223 | 2.71E-05 |
|  | *GFRA1* | 1.876 | 4.84E-04 |
|  | *LOC105371893* | 1.781 | 1.91E-03 |
|  | *SOWAHA* | 1.765 | 1.65E-03 |
|  | *RN7SL471P* | 1.756 | 2.18E-03 |
| **Downregulated in SRR AA compared to EA** | *MTRNR2L10* | -4.944 | 7.07E-32 |
|  | *MTRNR2L6* | -4.731 | 1.14E-36 |
|  | *RNU6-7* | -4.486 | 1.94E-38 |
|  | *RP11_161H235* | -4.414 | 1.67E-30 |
|  | *RN7SKP48* | -4.236 | 1.58E-67 |

**Table S2. Top differentially expressed genes from SRR residual tumor analysis.**

|  | **Ensembl ID** | **Gene** | **Log_2_Fold Change** | ***p* Value** |
| --- | --- | --- | --- | --- |
| **Upregulated in SRR AA compared to EA** | ENSG00000130810 | *PPAN* | 2.85 | 1.58 E-03 |
|  | ENSG00000117016 | *RIMS3* | 3.00 | 6.15 E-03 |
|  | ENSG00000117650 | *NEK2* | 3.03 | 1.13 E-02 |
|  | ENSG00000170075 | *GPR37L1* | 3.04 | 6.88 E-03 |
|  | ENSG00000105877 | *DNAH11* | 3.45 | 6.15 E-03 |
|  | ENSG00000204771 |  | 3.78 | 8.52 E-03 |
| **Downregulated in SRR AA compared to EA** | ENSG00000196754 | *S100A2* | -4.31 | 7.37 E-03 |
|  | ENSG00000227227 | *AC017101.1* | -3.61 | 6.88 E-03 |
|  | ENSG00000169604 | *ANTXR1* | -2.09 | 2.69 E-03 |
|  | ENSG00000204262 | *COL5A2* | -2.01 | 6.15 E-03 |
|  | ENSG00000072952 | *MRVI1* | -2.01 | 4.88 E-03 |
|  | ENSG00000269958 | *AL049840.4* | -1.36 | 4.73 E-02 |
|  | ENSG00000196663 | *TECPR2* | -1.17 | 1.21 E-02 |

**Table S3. Cohort Clinical Attributes.**

| **Clinical Attribute** | | **Treatment-Naïve** | | | | **Residual Tumor** | | | |
| --- | --- | --- | --- | --- | --- | --- | --- | --- | --- |
|  |  | **AA (*n* = 31)** | | **EA (*n* = 29)** | | **AA (*n* = 11)** | | **EA (*n* = 4)** | |
|  |  | **N** | **%** | **N** | **%** | **N** | **%** | **N** | **%** |
| **Stage** | **I** | 7 | 22.6 | 8 | 27.6 | 1 | 9.1 | 0 | 0.0 |
|  | **II** | 18 | 58.1 | 19 | 65.5 | 3 | 27.3 | 2 | 50.0 |
|  | **III** | 5 | 16.1 | 0 | 0.0 | 7 | 63.6 | 2 | 50.0 |
|  | **IV** | 1 | 3.2 | 2 | 6.9 | 0 | 0.0 | 0 | 0.0 |
| **Grade** | **II/III** | 1 | 3.2 | 2 | 6.9 | 3 | 27.3 | 1 | 25.0 |
|  | **III/III** | 32 | 96.8 | 29 | 93.1 | 8 | 72.7 | 3 | 75.0 |
