# Supplementary figures and images for: Identification of Distinct Heterogenic Subtypes and Molecular Signatures Associated with African Ancestry in Triple Negative Breast Cancer Using Quantified Genetic Ancestry Models in Admixed Race Populations
Source: Cancers (Basel). 2020 May 13;12(5):1220. doi: 10.3390/cancers12051220 (PMC7281131; doi:10.3390/cancers12051220)

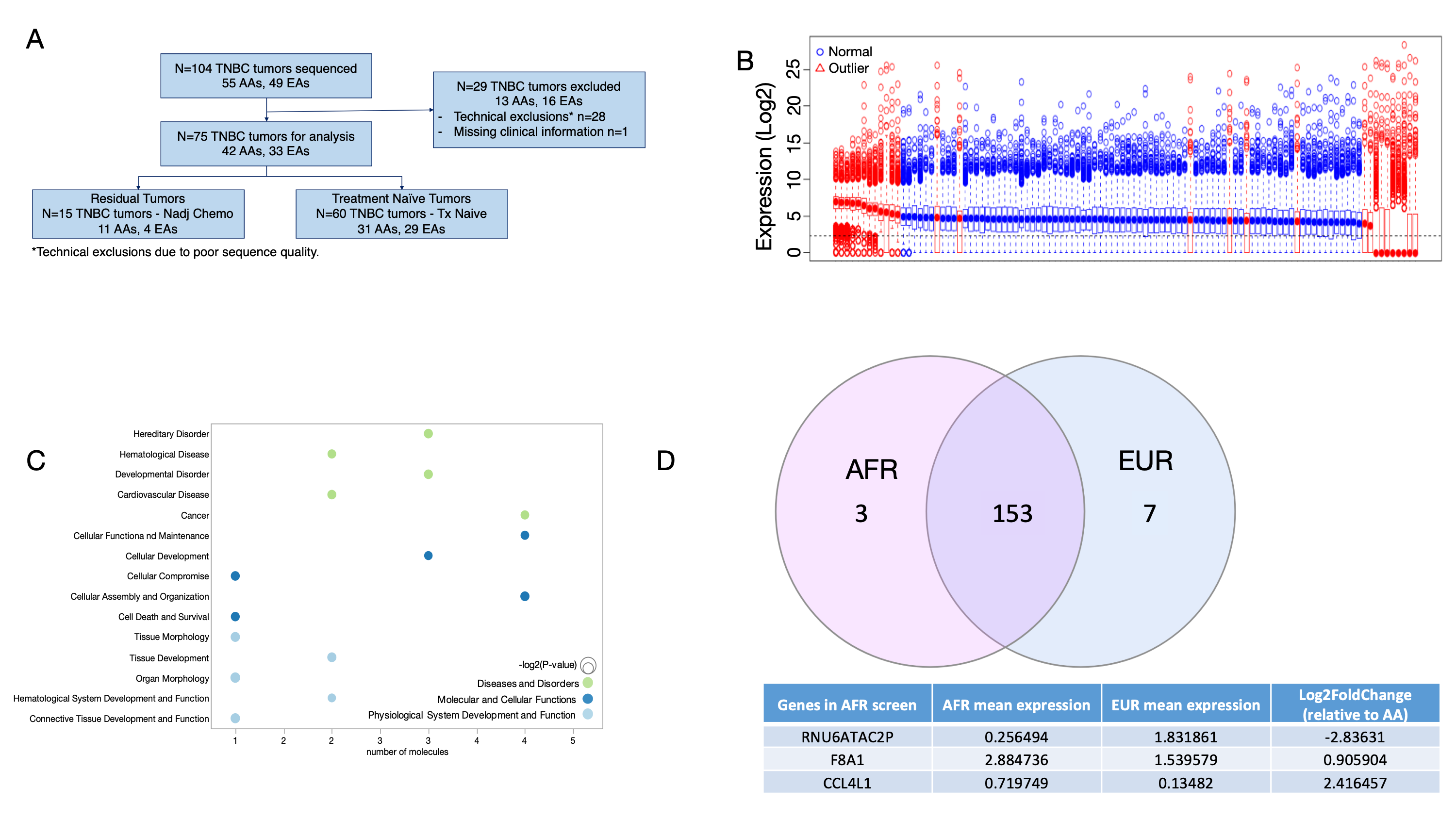

Supplement: Supplementary file 1 [file cancers-12-01220-s001.zip › cancers-785734 supplementary/Supplementary2020-04-29/SF 1.tiff]

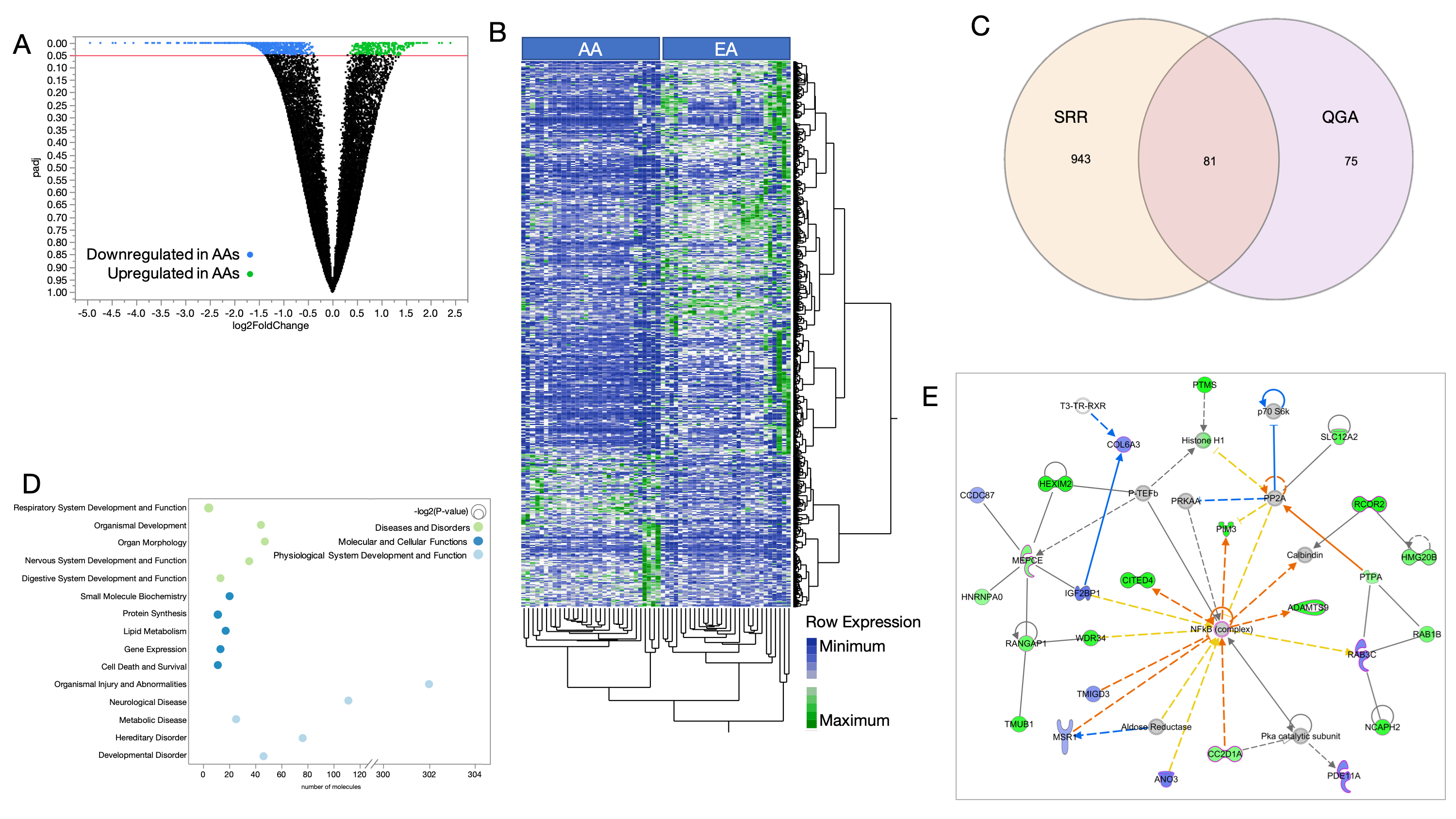

Supplement: Supplementary file 1 [file cancers-12-01220-s001.zip › cancers-785734 supplementary/Supplementary2020-04-29/SF 2.tiff]

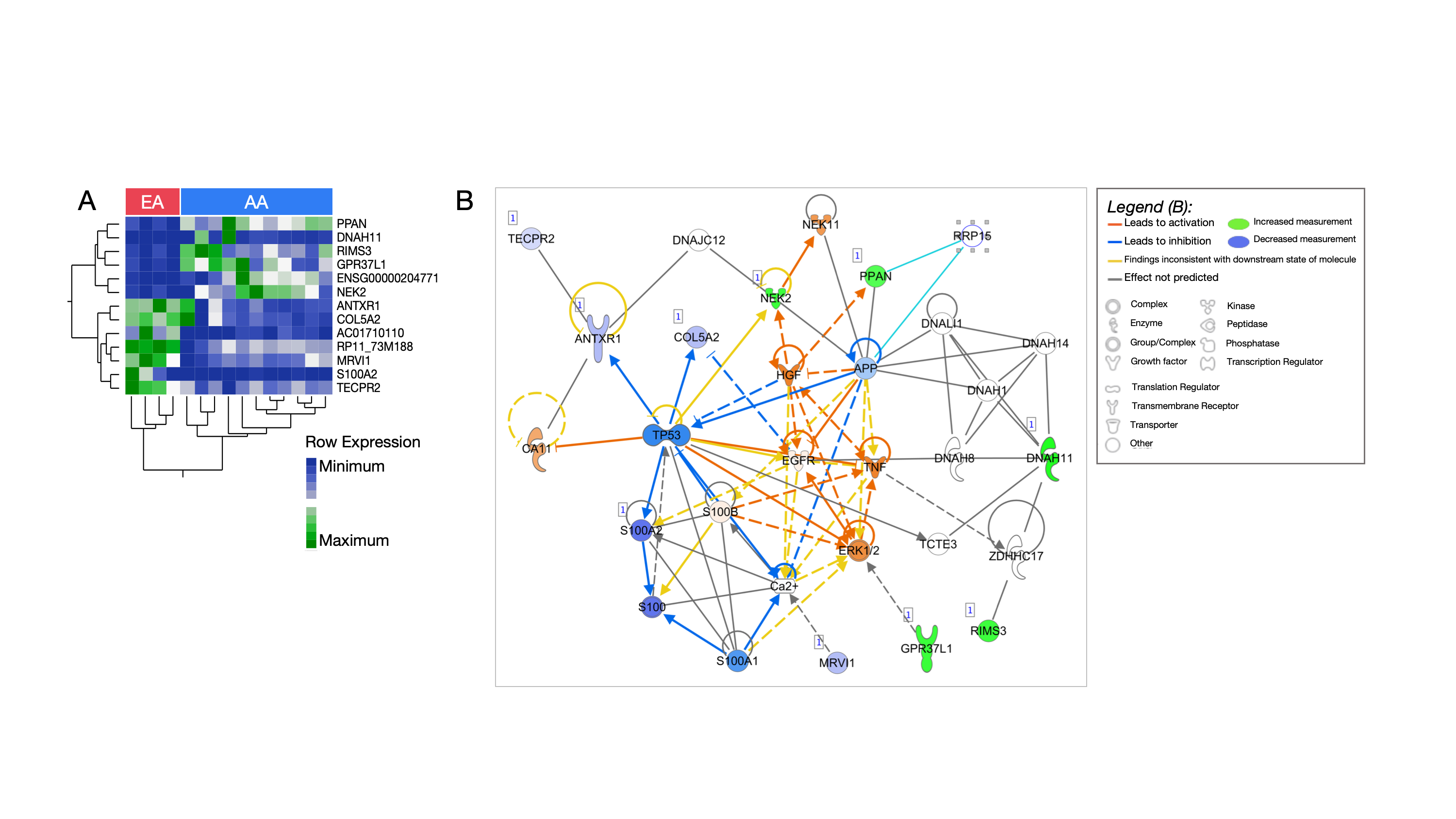

Supplement: Supplementary file 1 [file cancers-12-01220-s001.zip › cancers-785734 supplementary/Supplementary2020-04-29/SF 3.tiff]

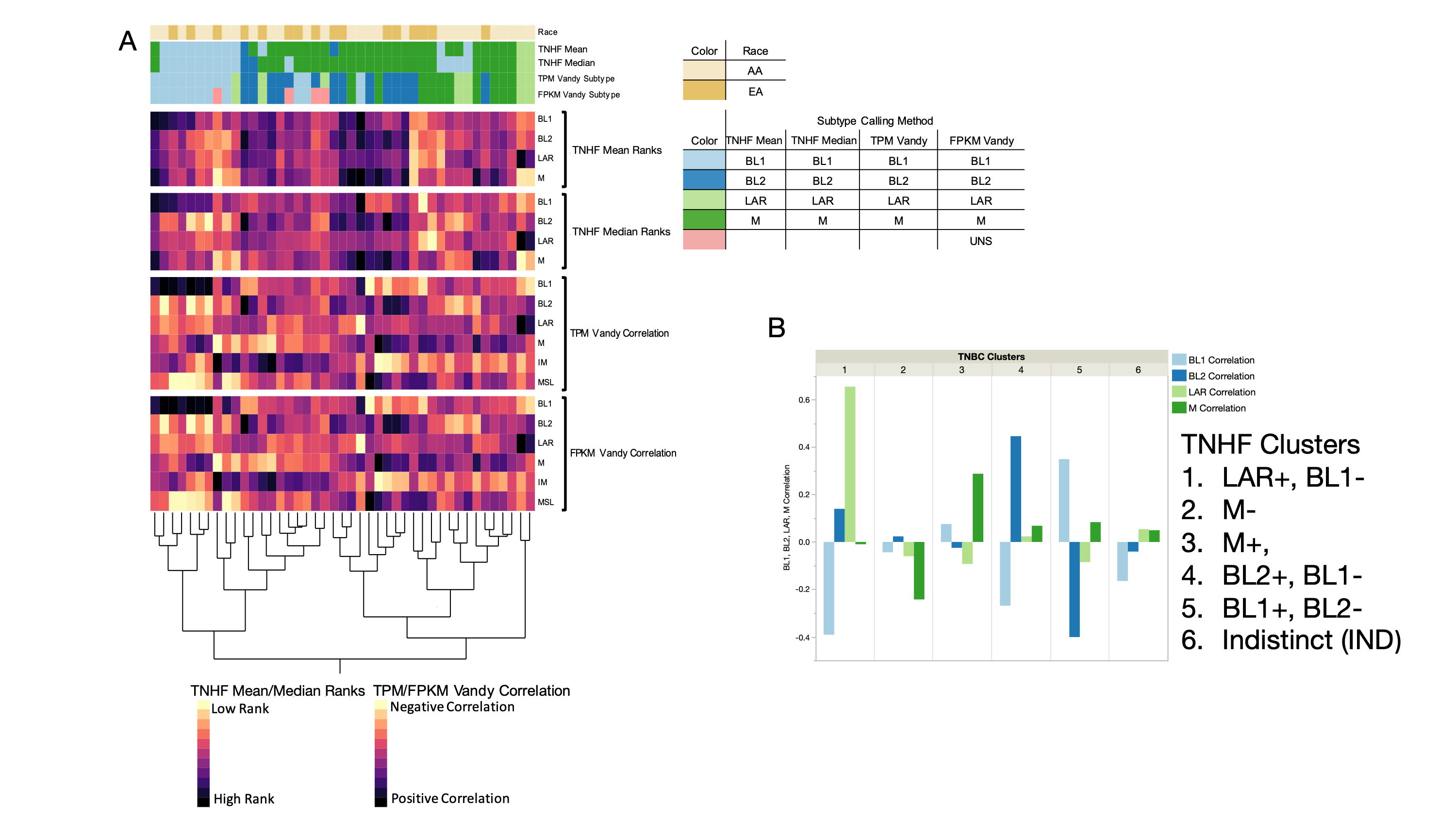

Supplement: Supplementary file 1 [file cancers-12-01220-s001.zip › cancers-785734 supplementary/Supplementary2020-04-29/SF 4.tiff]

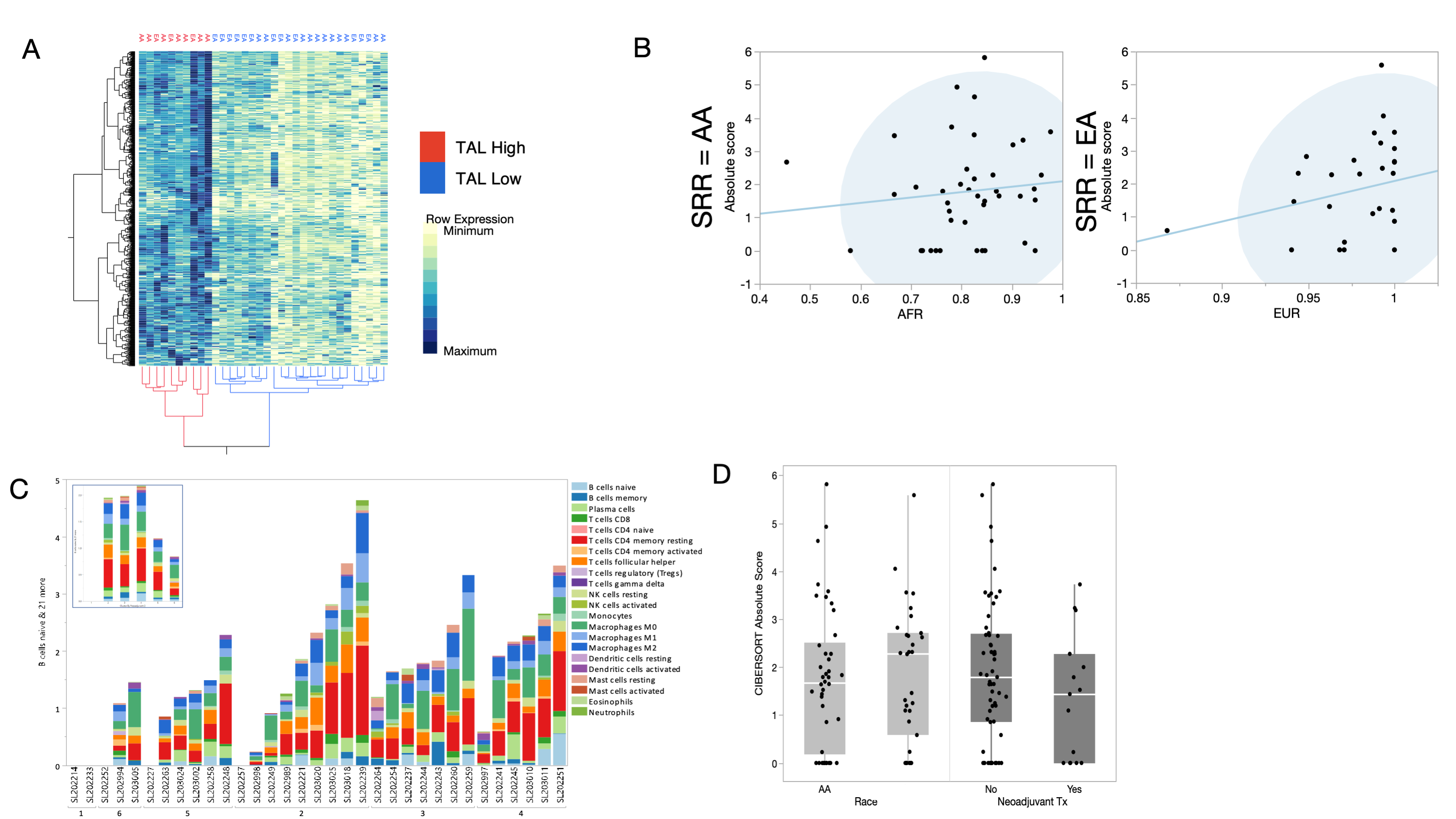

Supplement: Supplementary file 1 [file cancers-12-01220-s001.zip › cancers-785734 supplementary/Supplementary2020-04-29/SF 5.tiff]

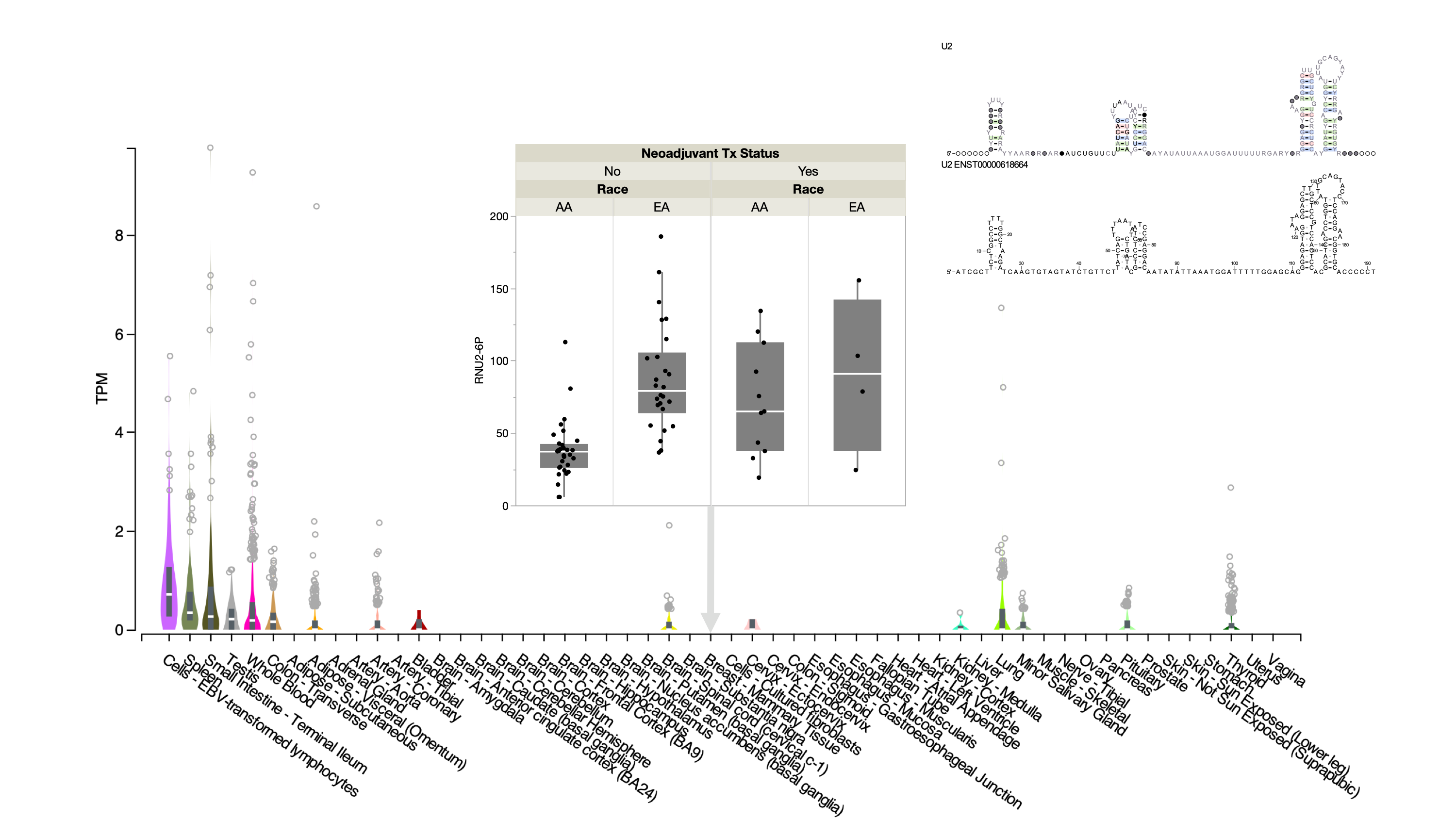

Supplement: Supplementary file 1 [file cancers-12-01220-s001.zip › cancers-785734 supplementary/Supplementary2020-04-29/SF 6.tiff]
